# 310. Yogurt Intake and Colorectal Cancer Incidence Subclassified by *Bifidobacterium S*tatus in Tumor

**DOI:** 10.1093/ofid/ofad500.382

**Published:** 2023-11-27

**Authors:** Satoko Ugai, Tomotaka Ugai, Shuji Ogino

**Affiliations:** Harvard T.H. Chan School of Public Health, Brookline, Massachusetts; Brigham and Women's Hospital, Brookline, Massachusetts; Brigham and Women's Hospital, Brookline, Massachusetts

## Abstract

**Background:**

Although epidemiological evidence is limited, experimental data indicated a tumor-suppressive effect of yogurt containing *Bifidobacterium*. We hypothesized that the association between yogurt intake and colorectal cancer incidence differed by *Bifidobacterium* status in tumor tissue.

**Methods:**

We utilized data from two prospective cohort studies: the Nurses’ Health Study and the Health Professionals Follow-up Study. Inverse probability weighted multivariable Cox proportional hazards regression was used to assess differential associations of yogurt intake with the incidence of colorectal carcinomas subclassified by tumor *Bifidobacterium* status.

**Results:**

During follow-up of 132,056 individuals, we documented 2,950 incident colorectal cancer cases, including 1,191 with available *Bifidobacterium* data. The association between yogurt intake and colorectal cancer incidence differed by *Bifidobacterium* status (*P*_heterogeneity_ = 0.03). This differential association became stronger in a subgroup analysis of proximal colon cancer (*P*_heterogeneity_ = 0.003). Multivariable hazard ratios in individuals who consumed ≥ 2 servings/week (vs. < 1 serving/month) of yogurt were 0.43 [95% confidence interval (CI), 0.22-0.85] for *Bifidobacterium*-positive proximal colon cancer, and 1.40 (95% CI, 0.95-2.05) for *Bifidobacterium*-negative proximal colon cancer.

**Conclusion:**

Yogurt intake was inversely associated with the incidence of *Bifidobacterium*-positive (but not *Bifidobacterium*-negative) proximal colon cancer, suggesting an interactive influence of yogurt intake and intestinal microbiota on tumor development.

**Disclosures:**

**All Authors**: No reported disclosures

